# Human-Inspired Reflex to Autonomously Prevent Slip of Grasped Objects Rotated with a Prosthetic Hand

**DOI:** 10.1155/2018/2784939

**Published:** 2018-06-24

**Authors:** Zachary Ray, Erik D. Engeberg

**Affiliations:** Florida Atlantic University, Boca Raton, FL 33431, USA

## Abstract

Autonomously preventing grasped objects from slipping out of prosthetic hands is an important feature for limb-absent people since they cannot directly feel the grip force applied to grasped objects. Oftentimes, a satisfactory grip force in one situation will be inadequate in different situations, such as when the object is rotated or transported. Over time, people develop a grip reflex to prevent slip of grasped objects when they are rotated with respect to gravity by their natural hands. However, this reflexive trait is absent in commercially available prosthetic hands. This paper explores a human-inspired grasp reflex controller for prosthetic hands to prevent slip of objects when they are rotated. This novel human-inspired grasped object slip prevention controller is evaluated with 6 different objects in benchtop tests and by 12 able-bodied subjects during human experiments replicating realistic tasks of daily life. An analysis of variance showed highly significant improvement in the number of successfully completed cycles for both the benchtop and human tests when the slip prevention reflex was active. An object sorting task, which was designed to serve as a cognitive distraction for the human subjects while controlling the prosthetic hand, had a significant impact on many of the performance metrics. However, assistance from the novel slip prevention reflex mitigated the effects of the distraction, offering an effective method for reducing both object slip and the required cognitive load from the prosthetic hand user.

## 1. Introduction

Approximately 541,000 people in the USA are living with an upper limb loss [1]; however, only 30%–50% of amputees use an electromyogram- (EMG-) controlled prosthetic hand or arm [2]. This high rejection rate is often because commercially available prostheses do not effectively solve problems for many limb-absent people, not because they are unavailable to them [3]. There is still a significant difference between prosthetic and human hands. This is due in no small part to the fact that the skin on the human hand has numerous sensory receptors which provide feedback to the central nervous system. These include the fast responding Pacinian and Meissner's corpuscles and the slow responding Ruffini corpuscle and Merkel cells. Each has unique sensory functions including the detection of vibration frequency, object texture, and finger pose, as well as grasp stability and force to name a few [4]. They provide highly efficient neural feedback allowing for a 0.06–0.08 second response to the onset of the object slip [5]. Replicating the functionality and autonomous control of a human hand with modern-day prostheses is a challenging task.

Most powered prosthetic hands, such as the Motion Control Hand (MCH), currently used have a single degree of freedom (DOF) to enable a three fingered pinch grasp. However, there have been great advances recently toward more dexterous prostheses, such as the Vincent hand (Vincent Systems), the Bebionic hand (RSL Steeper), and the i-limb (Touch Bionics) [6].

Powered prosthetic hands are often controlled using a set of EMG preamplifiers placed on antagonistic muscles [7]. EMG signals are typically band-pass filtered, rectified, and amplified to obtain a functional motor control signal where the muscle contraction controls the force or speed of the hand [8, 9]. Although EMG control is a well-established technique used for the actuation of prostheses, improvements must be made in order to lessen the need for the user's visual attention and the cognitive control burden [9, 10].

Many myoelectric prosthetic hands have a powered wrist joint for pronation and supination [11]. While controlling the wrist joint, clinical practice does not allow the user to simultaneously control the grip force of grasped objects. A majority of clinical upper limb prostheses in use today are operated open loop [12], which can lead to frustrating situations where objects are inadvertently dropped as the user cannot directly feel if there is a sufficient grip force to prevent slip as the object is being rotated by the wrist. Even if the operator could visually determine if the grip force was insufficient [13], it would be difficult to react quickly enough to halt slip after the grasped object begins to slide due to EMG filter time constants that are prevalent in clinically available prosthesis control schemes [2].

Grasped object slip prevention is important for prosthetic hands because the user has no direct sense of the applied grip force, making it common to inadvertently drop objects [14, 15]. There are two main approaches to autonomously prevent grasped objects from being accidentally dropped: reactive and proactive. In reactive slip prevention, specialized tactile sensors [16, 17] can be used to detect when a grasped object slips and the grip force can be autonomously increased to prevent the object from being dropped [18, 19]. With proactive slip prevention, as incorporated within the SensorHand Speed [20], risky situations can be identified that are likely to induce slip and the grip force is autonomously increased prior to the onset of slip. These scenarios include unfavorable grip force to load force ratios [20, 21] or increased velocity [22] and acceleration [23] of the wrist, and both of which are likely to destabilize the grasp safety margin and cause objects to be dropped.

Commonly used objects, such as tools, beverages, and personal items, require grip force compensation to prevent slip when rotated with respect to gravity [24]. For example, when an object is grasped with a human hand and pronated such that the grip axis is aligned with gravity, the object is likely to slip as the shift in the object's center of mass location creates a different torque at the fingertips. However, this trait is absent in prosthetic hands and could be problematic when limb-absent operators rotate grasped objects with a powered wrist or their residual limb.

Because limb-absent people have mentioned that autonomous slip prevention is a desirable trait for prosthetic hands (Table 2 in [14]), the focus of this paper is on the development of a novel proactive slip prevention controller. The human-inspired trait of autonomously increasing the grip force as grasped objects are rotated with respect to gravity [25] will be implemented within a hybrid force-position sliding mode controller [26]. Results from benchtop experiments using the human-inspired slip prevention controller reliant upon hand orientation feedback (HOF) with respect to gravity while grasping six different objects with the MCH are presented [27]. New additions to this paper over that previously presented [27] include data from 12 able-bodied subjects who used the Motion Control Hand with and without HOF during an object sorting task.

## 2. Prosthetic and Robotic Systems

### 2.1. The Motion Control Hand

The Motion Control Hand (Motion Control, Inc. Salt Lake City, USA) has a single DOF. It is instrumented with an A1321 Hall effect sensor (Allegro Micro Systems Inc., Worcester, USA) used to measure the distance between the thumb and forefingers which are connected via a motor-driven four-bar linkage. Strain gauges on the thumb measure normal force (*F*_N_) of the grasp. The hand is also equipped with a gyro (IDG-300, InvenSense, Inc., Santa Clara, CA, USA), which is used to measure the orientation of the wrist with respect to gravity.

State space equations [28] to describe the single DOF MCH are given by

(1) $$\begin{array}{c} {\dot{x}}_{1}=x_{2}, \end{array}$$

(2) $$\begin{array}{c} {\dot{x}}_{2}=-\frac{B}{J}x_{2}-\frac{K}{J}\left(x_{1C}-x_{1}\right)+\frac{n}{J}E-\frac{D}{J}, \end{array}$$

where *x*_1_ is the distance between the fingertips, *x*_1C_ is the position when the MCH makes contact with a given object, and *x*_2_ is the velocity. *E* is the voltage input, and *J* is the inertia of the system. *K* and *B* are the combined stiffness and damping of the grasped object-hand system, respectively; *n* is a constant derived from the gear ratio, armature resistance, and torque constant of the motor. *D* is the cumulative unknown and potentially nonlinear disturbances affecting the system.

### 2.2. Yaskawa SIA10F Robotic Arm

Motoman's SIA10F is a seven DOF robotic arm to which the Motion Control Hand is attached. Only the distal joint of the arm was necessary for this study to simulate human pronation and supination of the wrist as described in [25]. The SIA10F robotic arm utilizes the FS100 controller and DX100 Teach Pendant.

## 3. Sliding Mode Controller

Sliding mode control (SMC) has been implemented for prosthetic hands in the past using a hybrid force-position control law [29], which is particularly useful for prosthetic hands because it facilitates an ability to control both the force and the position of the hand through a single input. When grasping an object, the desired force from the operator is *F*_D_, which is realized using an outer force control loop to form a force error signal. This force error signal yields the desired position of the hand:

(3) $$\begin{array}{c} x_{D}=G_{F}\left(F_{D}-F_{N}\right). \end{array}$$

This force error, shown as the difference between *F*_D_ and the measured normal force of the hand *F*_N_, is scaled by the gain, *G*_F_. To enable sliding mode control, a position error is next formed as

(4) $$\begin{array}{c} e=x_{D}-x_{1}, \end{array}$$

with which a sliding manifold is formed as

(5) $$\begin{array}{c} S=G_{P}e+G_{D}\dot{e}, \end{array}$$

where *G*_P_ is the proportional gain and *G*_D_ is the derivative gain. This enables control over the applied grip force as well as the position of the hand even if an object is not grasped.

**Figure 1:**
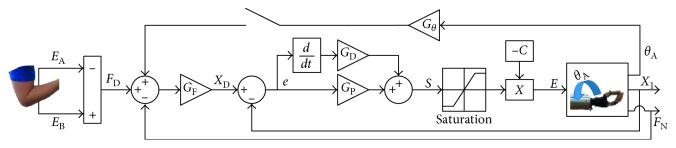
Diagram of the sliding mode controller. When the upper loop is closed, hand orientation feedback modulates the grip force.

The sliding mode controller (Figure 1) has been demonstrated to be robustly stable using the following control law:

(6) $$\begin{array}{c} E=-C\text{ sat}\left(S\right). \end{array}$$

The constant, C, is based on an upper bound estimate on the torques acting on the motor of the hand and sat represents the saturation function to partially linearize the controller and prevent undesirable chatter or oscillations. Refer [26] for more details about this controller and [30] for discussion about the stability of sliding mode control for a broad class of systems.

## 4. Human-Inspired Reflexive Slip Prevention Controller

Based on prior research, it is clear that the human grip force is coupled to wrist motions to maintain grasp stability [31]. A human-inspired prosthetic hand control strategy will be developed in this paper to mimic this trait. Prosthetic hand orientation feedback will be used to impart the anthropomorphic trait of modulating the grasp force based on pronation and supination motions of the wrist with respect to gravity, which had been studied in people [25]. This is a proactive slip prevention technique that is used to increase the grasp force when the grip axis is rotated through the field of gravity so that grasped objects are not inadvertently dropped. The specific control mechanism to enable this biomimetic trait is to feed back the measured wrist angle into the outer force feedback loop so that the desired position becomes

(7) $$\begin{array}{c} x_{D}=G_{F}\left(F_{D}-F_{N}+G_{\theta }\theta_{A}\right). \end{array}$$

The orientation of the hand with respect to gravity is denoted by *θ*_A_ in rad, and *G*_*θ*_ is a proportional gain. With the inclusion of this positive feedback term, the applied grip force is increased relative to the rotation of the wrist relative to gravity, to mimic the human trait of proactive slip prevention during wrist rotation [25]. This sliding mode controller with HOF is shown in Figure 1 when the upper switch is closed.

Note that this HOF controller is robustly stable since the error term is still minimized by the sliding mode controller (6); the wrist angle feedback can be thought of as an autonomous modifier of the hand operator's desired force signal. This is useful to reduce the cognitive burden required to operate prosthetic hands as will be subsequently shown.

## 5. Experimental Methods

The sliding mode controller is implemented using Simulink (MathWorks, Natick, USA) and the real-time windows target kernel. Data were recorded at a rate of 1 kHz.

**Figure 2:**
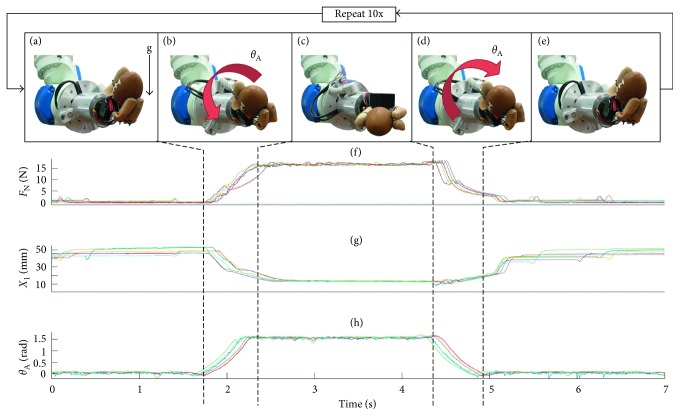
(a–e) Pronation-supination motion sequence aligned with (f) normal force, (g) fingertip distance, and (h) wrist angle.

For each experiment, the hand is initially set to grasp a given object, with the grip axis in the plane of gravity (Figure 2). Once grasped, the hand pronates *π*/2 rad in 0.5 seconds (Figure 2). The hand remains oriented with the grip axis perpendicular to gravity for 2 seconds (Figure 2). It then supinates back to the start position with the grip axis in line with gravity in 0.5 seconds (Figure 2). The hand remains in this position with the grip axis in line with gravity (Figure 2) for 2 seconds, at which time the entire cycle (Figures 2–2) is repeated by the Yaskawa arm according to a predetermined program.

### 5.1. Benchtop Tests

**Figure 3:**
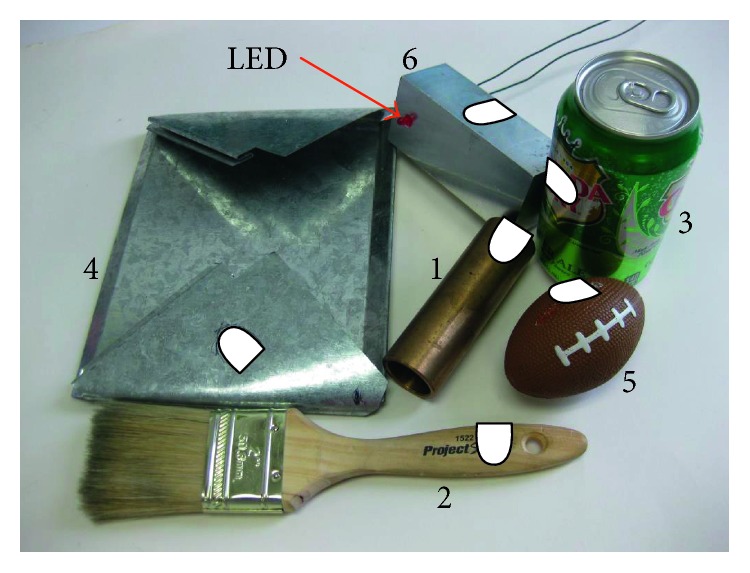
The grasped and rotated items includes (1) copper tube (262 g), (2) paintbrush (57 g), (3) full soda can (386 g), (4) compliant sheet metal (164 g), (5) stress football (25 g), and (6) aluminum block (461 g) equipped with a LED to indicate if the object was squeezed too tightly during the human trials. The superimposed white thumbprint shows how the item was grasped.

Six relatively common items numbered one through six in Figure 3 were used in this study. The figure shows the grasp location of the thumb for each grasped item represented by the superimposed white thumbprint. The copper tube (Object 1, 262 g) was grasped at one end to induce a noticeable gravitational torque when the grip axis was rotated out of the plane of gravity. The grasp location for the paintbrush (Object 2, 57 g) was its wooden handle. The sealed aluminum soda can (Object 3, 386 g) was grasped around its middle. The compliant scrap metal (Object 4, 164 g) was used to show how the control system reacted to a deformable object. The scrap metal had a stiffness of 2.4 N/mm over the range of deformations imparted in this study. The compliant foam football (Object 5, 25 g) had a stiffness of 0.47 N/mm. The aluminum block (Object 6, 461 g) was also tested prior to use by the human subjects.

Each of the six objects was subjected to two different benchtop tests. The first test involved observing how the MCH performed the pronation/supination task without the influence of HOF. Each object was grasped with the minimum grip force and then subjected to the predefined rotations (Figures 2–2). The second test was identical to the first but with the HOF included (7) by closing the top feedback loop shown in Figure 1. Each test was repeated for ten trials, and each trial consisted of ten possible pronation/supination cycles. The cycle count stopped if the object was dropped.

The effect of each object and the use of the human-inspired HOF on the number of successful cycles completed were analyzed using a two-factor ANOVA test.

### 5.2. Human Trials

Twelve able-bodied subjects (four females and eight males) participated in this experiment. All subjects gave voluntary written and informed consent in accordance with the approved IRB protocol.

Each subject was allowed approximately 15 minutes to familiarize him or herself with EMG control while the experimenter calibrated the EMG hardware (MyoLab II, Motion Control, Inc. Salt Lake City, USA) for each individual. The subject sat comfortably in an office chair facing the prosthetic hand with the EMG preamplifiers strapped to the forearm of his or her nondominant hand. One preamplifier was placed atop the extensor digitorum communis muscle, and the other preamplifier was placed over the flexor carpi radialis [32].

**Figure 4:**
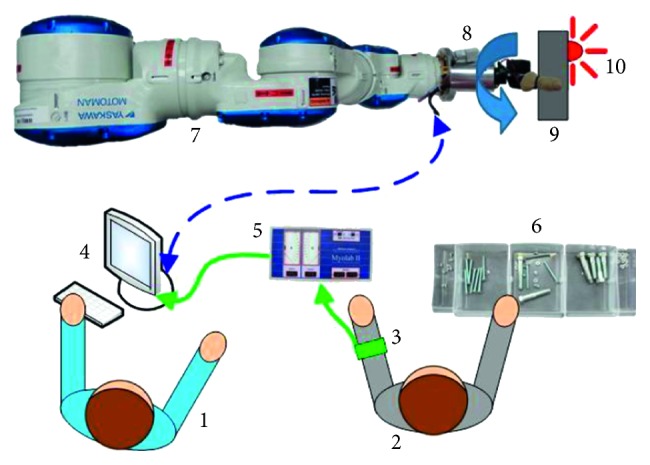
Test environment: (1) test operator; (2) test subject; (3) EMG preamplifiers strapped to the subject's forearm; (4) computer running Simulink; (5) MyoLab II for EMG signal processing; (6) sorting task; (7) Yaskawa seven DOF robot arm; (8) Motion Control Hand; (9) aluminum block, Object 6 in Figure 3; (10) LED indicator showing a failure if the object was squeezed too tightly.

The dominant hand was kept free for a sorting task performed in the second half of this experiment. This sorting task served as an additional cognitive load that is similar to sorting tasks performed daily; it consisted of separating a mix of four types of nuts and bolts (50 pieces total) into unique containers.  Figure 4 shows a diagram of the testing environment including the data flow for the EMG to the DAQ (green) and signals sent to and from the robot (dashed blue). All subjects were timed as they completed the sorting task three times prior to EMG experimentation. This baseline test provided information on the individual's sorting rate while unhindered by the additional task of EMG control.

All subjects participated in four different sets of experiments with the MCH grasping the instrumented aluminum block (Figure 3, Object 6). Each of the four tests was repeated for three trials, and each trial consisted of ten possible pronation/supination cycles. The total number of completed cycles depended on the subject's success rate. The first two tests performed by all subjects were either EMG control without HOF or EMG control with HOF. The third and fourth tests were the same as the first two; however, the subjects in these cases were also asked to simultaneously perform the previously mentioned nuts and bolts sorting task. The twelve subjects were separated into one of the four different groups (three subjects per group) and performed each experimental condition in different orders to counterbalance the impact of learning with the different control configurations and tasks (Table 1).

Two failure conditions were possible for each test: a break condition and a drop condition. The instrumented aluminum block (Figure 3, Object 6) used in this study was equipped with an LED which lit up if the break condition force threshold was surpassed. The strain gauges in the MCH's thumb were used to determine the normal force applied to the object. The normal force for the break condition threshold was set to offer a moderate challenge while rotating the object. The number of breaks per trial was recorded in Simulink, but the testing continued regardless of break failures. If the object was dropped, the drop failure condition was tallied and the failed trial was terminated. The outline of the MCH's thumb was traced onto the block, and it was also considered a “drop” failure if the object slipped out of the traced area. During the third and fourth tests involving the sorting task, the number of nuts and bolts correctly sorted was also recorded for each trial from which the average rate of sorting was calculated.

After completing the experiments, each person was also asked to subjectively rate the difficulty of each of the four experimental combinations with and without being required to sort objects with or without HOF. A scale of 1 to 10 was used with 1 being difficult and 10 being easy.

The statistical significance of individual subject performance, HOF, and the sorting task on the collected data for number of successful cycles, drops, and breaks was analyzed using a three-factor ANOVA test. Also, the effect of variance caused by subject performance and HOF on the sorting count and sorting rate was analyzed using a two-factor ANOVA test. These analyses were performed to ascertain whether HOF with or without the cognitive load from the sorting task significantly impacted the performance metrics and whether or not there was interaction among any of the factors. Statistical significance of the subjective ratings was analyzed using a nonparametric Wilcoxon rank sum test for equal medians.

## 6. Results

### 6.1. Benchtop Tests

The data plots of Figure 2 illustrate the grip force, *F*_N_, the distance between the MCH's fingers, *x*_1_, and the angle of the wrist, *θ*_A_. The normal force (Figure 2) applied to the grasped object increased to compensate for the wrist rotation with HOF. The tip-to-tip distance between the finger and thumb of the MCH (Figure 2) decreased as the compliant object deformed, but it remained nearly constant when grasping rigid objects. The wrist pronated and supinated through the *π*/2 radians (Figure 2) in 0.5 seconds.

**Figure 5:**
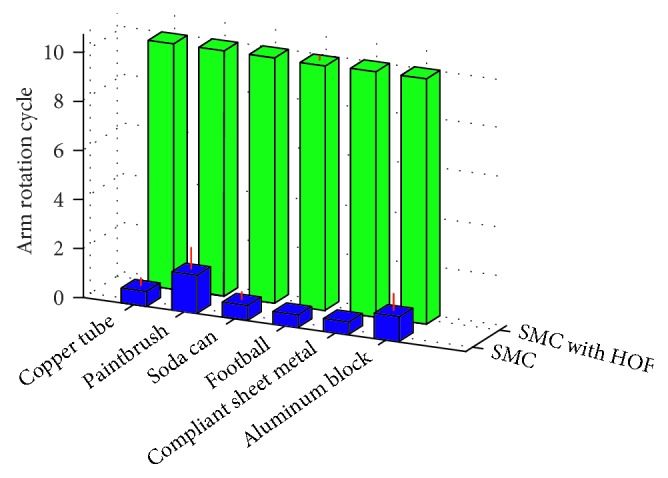
The number of successful cycles completed using the SMC without HOF is shown in blue, while the number of successful cycles completed using the SMC with HOF is shown in green for each object tested. Red lines indicate the standard deviations.

Objects grasped by the MCH without HOF were most frequently dropped on the first or second cycle. The objects grasped with HOF remained in the hand for all cycles with the exception of one football rotation cycle (Figure 5). The variance in the number of successful cycles completed was significantly impacted by the unique object and HOF (*p* < 0.01), but not their interaction (*p* > 0.05). The overall average number of successful cycles completed for each object was 0.79 ± 0.37 for SMC and 9.99 ± 0.03 for SMC with HOF.

### 6.2. Human Subject Results

**Figure 6:**
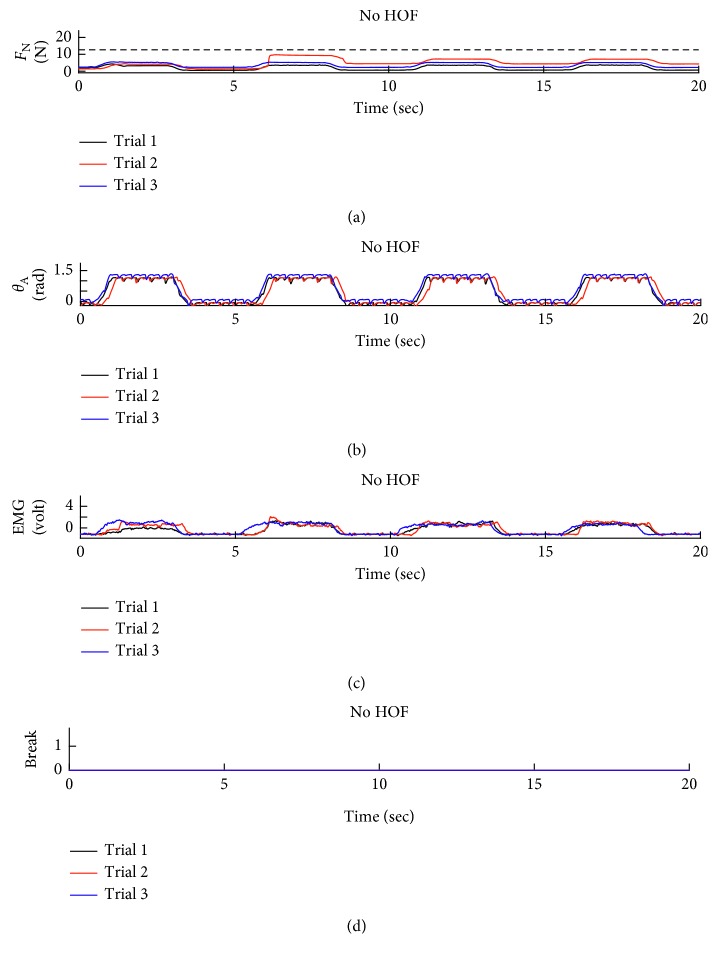
Test data for Group 2, Subject 1. This dataset shows the subject using EMG to control the hand. This subject was able to maintain a steady grip while focused on the task at hand.

**Figure 7:**
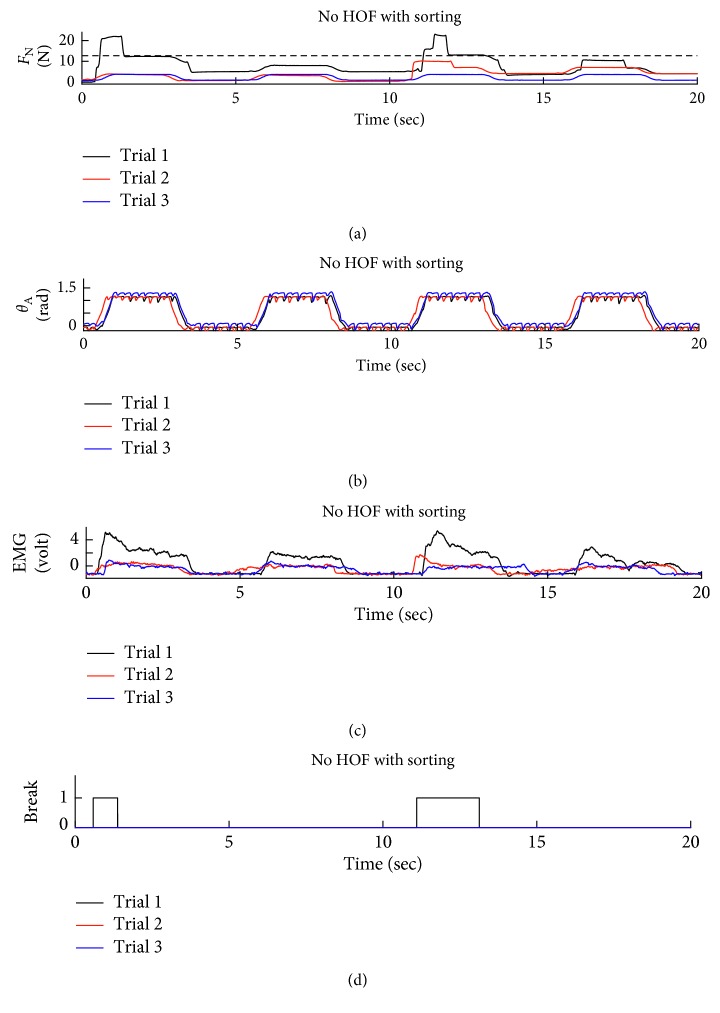
Test data for Group 2, Subject 1. This dataset shows the subject using EMG to control the hand without the assistance of HOF while simultaneously sorting parts. This subject was unable to maintain the same level of focus resulting in the grasp force exceeding the dashed line which represents the break threshold.

Sample data for two different tests are presented for a subject with a relatively high level of skill with EMG control of the prosthetic hand in Figures 6 and 7. The first two subplots in each figure show the normal force and wrist angle similar to the benchtop tests. The dashed line in the normal force subplots shows the break failure threshold. Notice that this threshold is not crossed in Figure 6 like it is in Figure 7. The additional cognitive load represented by the sorting task is apparent in the EMG signals of Figures 6 and 7 as the subject is unable to focus entirely on a single task. An example of a break failure is recorded as shown in Figure 7. The EMG input signals for each trial are included in these figures. These signals show a nearly proportional relationship between the EMG signal and the normal force the hand applies to the object.

**Figure 8:**
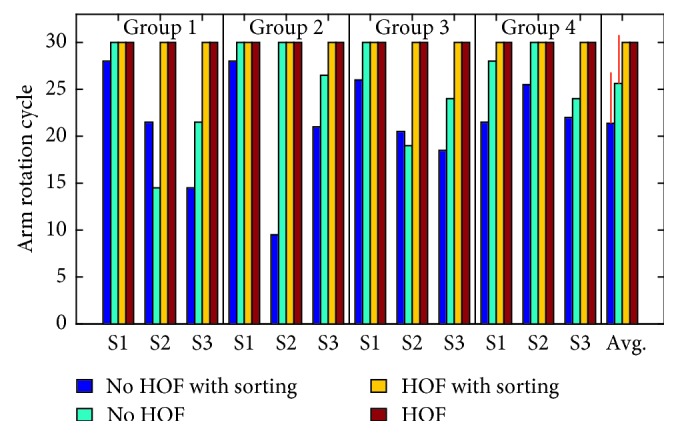
Total number of successful cycles out of 30 (10 for each of the 3 trials) attempted trials. S1, S2, and S3 are the first, second, and third subjects in each of the four groups.

**Figure 9:**
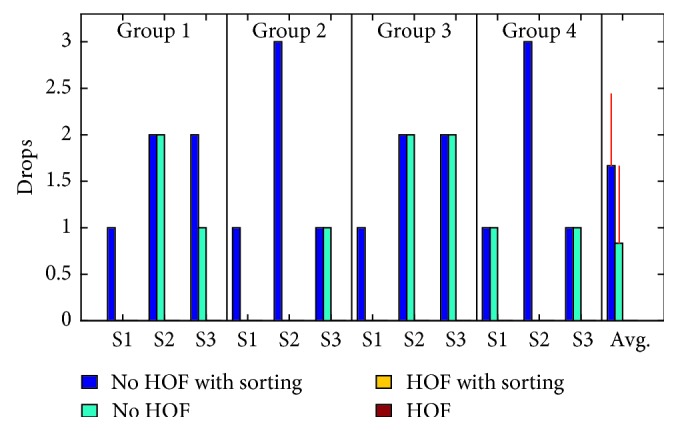
Total number of drop failures for each test out of 3.

The number of successfully completed cycles shown in Figure 8 shows the efficacy of the artificial slip prevention reflex afforded by HOF. Each test had a maximum of three possible drop failures, and the total number of drops is shown in Figure 9. In these figures, S1, S2, and S3 are the first, second, and third human subjects in each of the four groups. The overall average and standard deviation for the number of successfully completed cycles and number of drops is shown in Table 2. The sorting task had a significant impact on the number of drops and total number of successful cycles, but the HOF significantly improved this metric (Table 3).

**Figure 10:**
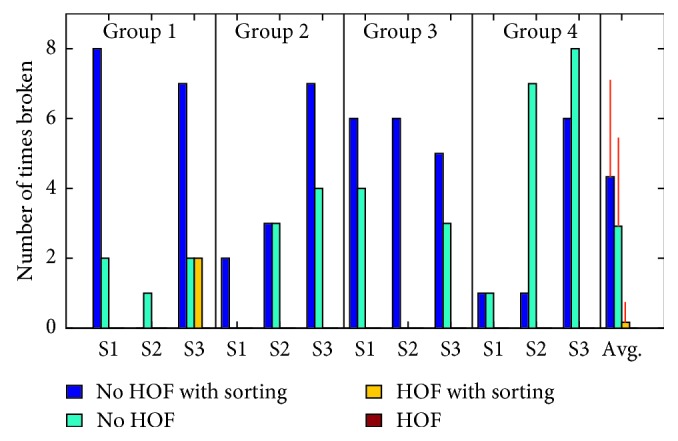
Total number of break failures for the 12 individual subject's successful number of cycles out of 30.

The maximum possible number of break failures for each test was 30 (three trials with ten pronation/supination cycles each) if the object was not dropped.  Figure 10 shows the total number of break failures by each subject for every test. The overall average and standard deviation for number of break failures is shown in Table 2. The total break count was not significantly impacted by the sorting task, but it was significantly improved with the use of HOF (Table 3).

**Figure 11:**
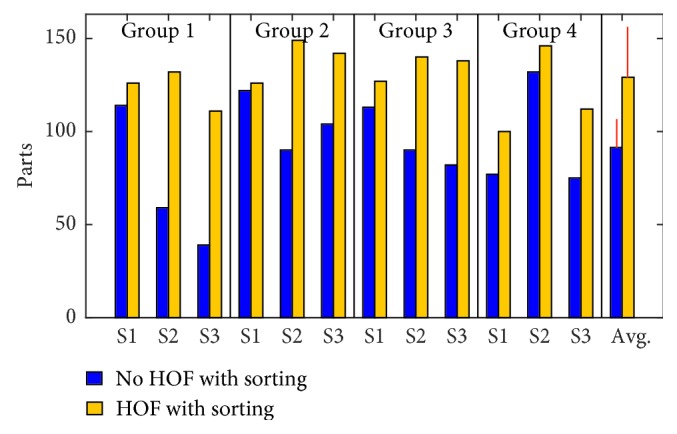
Total number of parts sorted out of 150 (50 for each of the 3 trials).

The number of parts sorted and the completion time for the sorting task were recorded and compared to the baseline case when the subjects sorted the nuts and bolts prior to controlling the hand. The average sorting rate was calculated from three iterations of the sorting task for each subject to serve as a baseline comparison to the sorting rate obtained while controlling the hand with and without HOF. The total number of parts sorted was summed for each of the three trials performed with and without HOF (Figure 11). The average sorting rate was then determined based on the number of parts sorted and the duration of the successful cycles for each trial. The overall average and standard deviation for these is shown in Table 2. It is clear that more parts were sorted with HOF and the sorting rate was fairly consistent. An ANOVA test reveals that the total number of parts sorted was significantly more with HOF (Table 3) because the objects were not dropped meaning that the subject had the maximum possible amount of sorting time. The influence of the subject was insignificant (Table 3). The sorting rate was different in the sense that HOF was not a significant factor, and the subject was a significant factor (Table 3). This can be attributed to the fact that each subject sorted at an individual pace of which the HOF was independent.

**Figure 12:**
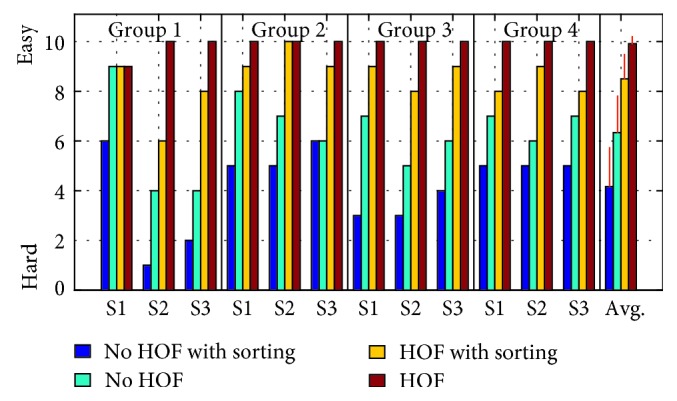
Subjective difficulty rating for each test scaling from 1 (hard) to ten (easy).

Subjects provided a qualitative rating of the difficulty for each test ranging from one (very difficult) to ten (very easy) shown in Figure 12. As expected, the sorting task increased the difficulty, but the tests performed without HOF were rated much more difficult than those with HOF. Overall averages and standard deviations for the subjective ranking are shown in (Figure 12). The order of the subjective ranking of the tests from easiest to hardest was statistically proven to be HOF without sorting, HOF with sorting, no HOF without sorting, and finally no HOF with sorting (Figure 12).

The order in which subjects attempted each test was structured to counterbalance the effect of a learning curve for the overall group of 12 subject's EMG operation of the MCH (Table 1). An ANOVA study showed the subject's influence on the data collected due to learning curve to be insignificant (Table 3).

## 7. Discussion

The increased use of sensor feedback will likely be more common in future prosthetic hand designs to allow more functional human-inspired closed loop control [33]. In this paper, both benchtop and human-controlled prosthetic hand experiments have demonstrated the utility of a novel grasped object slip prevention reflex enabled by HOF with respect to gravity for the MCH. Extension of the HOF slip prevention technique to multi-DOF hands such as the i-limb would be simple provided the forward kinematics equations were used to calculate the orientation of the grip axis. Another solution to circumvent the need for forward kinematics (which would require joint angle sensors) is to embed a small accelerometer into the distal link of the prosthetic finger to assess the grip axis orientation with respect to gravity. This would be useful for different grasp types such as power grip, precision grip, lateral pinch, and key grip [34]. It may also be useful to incorporate this control system into more complex hand synergies, similar to the one discussed in [7].

Even with advanced surgical procedures such as targeted muscle reinnervation [35], there will likely be less biocontrol signals available than controllable DOFs in the next generation of dexterous prosthetic hands such as the DEKA arm [35] and Modular Prosthetic Limb [36], both of which have powered wrists. Thus, there will be a continued need in the future for human-inspired low-level control algorithms [37, 38] such as the slip prevention reflex enabled by HOF to alleviate the operator's cognitive burden and reduce training time to gain proficiency.

## 8. Conclusion

The human-inspired grasped object slip prevention reflex enabled by hand orientation feedback dramatically improved the prosthetic hand's ability to maintain a precision grip on objects that were subjected to wrist pronation and supination. Benchtop tests showed the utility of the technique with six different objects with a wide range of mechanical characteristics. Human tests showed far fewer drop and break failures for each object and person with HOF. A realistic sorting task performed during testing showed the usefulness of HOF for all 12 human subjects, which was further corroborated in their qualitative controller evaluations. The object was broken and dropped much less frequently with the use of HOF while still sorting at approximately the same speed. Additionally, it would be easy to scale the technique to powered prosthetic elbows and shoulders. This human-inspired slip prevention reflex provides an inexpensive and practical way to anthropomorphically prevent grasped object slip while rotating objects, which would be very useful for prosthetic hands.

## Figures and Tables

**Table 1:** Testing order for the 12 subjects who were separated into four groups. The first two EMG tests were done without the sorting task, and the third and fourth EMG tests were done with the sorting task.

Group	Without sorting		With sorting	
	Test 1	Test 2	Test 3	Test 4
1	HOF	No HOF	HOF	No HOF
2	HOF	No HOF	No HOF	HOF
3	No HOF	HOF	HOF	No HOF
4	No HOF	HOF	No HOF	HOF

**Table 2:** The overall average ± standard deviation of each performance metric for each of the four tests.

Performance metric	Without HOF with sorting	Without HOF without sorting	With HOF with sorting	With HOF without sorting
Successful cycles	21.4 ± 5.43	25.6 ± 5.15	30.0 ± 0.00	30.0 ± 0.00
Drop count	1.67 ± 0.778	0.833 ± 0.835	0.000 ± 0.000	0.000 ± 0.000
Break count	4.33 ± 2.77	2.92 ± 2.54	0.167 ± 0.577	0.000 ± 0.000
Sort count	91.4 ± 27.1	—	129 ± 15.2	—
Sort rate	0.819 ± 0.187	—	0.870 ± 0.115	—
Subjective ranking	4.17 ± 1.59	6.33 ± 1.50	8.50 ± 1.00	9.92 ± 0.289

**Table 3:** *p* values from the three-factor ANOVA showing the level of effect that the subject's performance, the HOF, and the sorting task had on the collected data shown in the columns. It is clear that HOF had a highly significant impact on all data except the sorting rate, which was primarily affected by the individual subject's performance. The sorting task had a significant impact on all data except the break count.

Variable	Successful cycles	Drop count	Break count	Break rate	Sort count	Sort rate
Subject	0.194	0.440	0.255	0.254	0.095	0.012
HOF	0.000	0.000	0.000	0.000	0.000	0.221
Sorting	0.043	0.025	0.131	0.029	—	—
Subject HOF	0.194	0.440	0.296	0.350	0.943	0.397
Subject sorting	0.500	0.500	0.368	0.347	—	—
HOF sorting	0.043	0.025	0.224	0.045	—	—
Subject HOF sorting	0.989	1.000	0.984	0.862	—	—

## References

[1] Ziegler-Graham K., MacKenzie E., Ephraim P., Travison T., Brookmeyer R. (2008). Estimating the prevalence of limb loss in the US: 2005-2050. *Archives of Physical Medicine and Rehabilitation*.

[2] Fougner A., Stavdahl O., Kyberd P., Losier Y., Parker P. (2012). Control of upper limb prostheses: terminology and proportional myoelectric control-a review. *IEEE Transactions on Neural Systems and Rehabilitation Engineering*.

[3] Biddiss E., Beaton D., Chau T. (2007). Consumer design priorities for upper limb prosthetics. *Disability and Rehabilitation: Assistive Technology*.

[4] Dahiya R. S., Metta G., Valle M., Sandini G. (2010). Tactile sensing—from humans to humanoids. *IEEE Transactions on Robotics*.

[5] Johansson R. S., Westling G. (1984). Roles of glabrous skin receptors and sensorimotor memory in automatic control of precision grip when lifting rougher or more slippery objects. *Experimental Brain Research*.

[6] Belter J., Segil J., Dollar A., Weir R. (2013). Mechanical design and performance specifications of anthropomorphic prosthetic hands: a review. *Journal of Rehabilitation Research and Development*.

[7] Kent B., Karnati N., Engeberg E. (2014). Electromyogram synergy control of a dexterous artificial hand to unscrew and screw objects. *Journal of NeuroEngineering and Rehabilitation*.

[8] De Luca C. J. (1997). The use of surface electromyography in biomechanics. *Journal of Applied Biomechanics*.

[9] Oskoei M., Hu H. (2007). Myoelectric control systems-a survey. *Biomedical Signal Processing and Control*.

[10] Cordella F., Ciancio A., Sacchetti R. (2016). Literature review on needs of upper limb prosthesis users. *Frontiers in Neuroscience*.

[11] Bajaj N., Spiers A., Dollar A. State of the art in prosthetic wrists: commercial and research devices.

[12] Saunders I., Vijayakumar S. (2011). The role of feed-forward and feedback processes for closed-loop prosthesis control. *Journal of NeuroEngineering and Rehabilitation*.

[13] Engeberg E., Meek S. (2012). Enhanced visual feedback for slip prevention with a prosthetic hand. *Prosthetics and Orthotics International*.

[14] Biddiss E., Chau T. (2007). Upper limb prosthesis use and abandonment: a survey of the last 25 years. *Prosthetics and Orthotics International*.

[15] Kyberd P., Wartenberg C., Sandsjo L. (2007). Survey of upper-extremity prosthesis users in Sweden and the United Kingdom. *Journal of Prosthetics and Orthotics*.

[16] Fishel J., Loeb G. (2012). Bayesian exploration for intelligent identification of textures. *Frontiers in Neurorobotics*.

[17] Vatani M., Engeberg E., Choi J. (2013). Force and slip detection with direct-write compliant tactile sensors using multi-walled carbon nanotubes/polymer composites. *Sensors and Actuators A: Physical*.

[18] Engeberg E., Meek S. (2013). Adaptive sliding mode control for prosthetic hands to simultaneously prevent slip and minimize deformation of grasped objects. *IEEE/ASME Transactions on Mechatronics*.

[19] Yamada Y., Morita H., Umetani Y. (2000). Slip phase isolating: impulsive signal generating vibrotactile sensor and its application to real-time object regrip control. *Robotica*.

[20] Puchhammer G. (2000). The tactile slip sensor: integration of a miniaturized sensory device on an myoelectric hand. *Orthopadie*.

[21] Wettels N., Parnandi A., Moon J., Loeb G., Sukhatme G. (2009). Grip control using biomimetic tactile sensing systems. *IEEE/ASME Transactions on Mechatronics*.

[22] Kent B., Engeberg E. (2017). Robotic hand acceleration feedback to synergistically prevent grasped object slip. *IEEE Transactions on Robotics*.

[23] Engeberg E., Frankel M., Meek S. Biomimetic grip force compensation based on acceleration of a prosthetic wrist under sliding mode control.

[24] Balasubramanian R., Xu L., Brook P., Smith J., Matsuoka Y. (2012). Physical human interactive guidance: identifying grasping principles from human-planned grasps. *IEEE Transactions on Robotics*.

[25] Johansson R., Backlin J., Burstedt M. (1999). Control of grasp stability during pronation and supination movements. *Experimental Brain Research*.

[26] Engeberg E. D., Meek S. G., Minor M. A. (2008). Hybrid force-velocity sliding mode control of a prosthetic hand. *IEEE Transactions on Biomedical Engineering*.

[27] Ray Z., Engeberg E. D. Hand orientation feedback for grasped object slip prevention with a prosthetic hand.

[28] Nise N. (2002). *Control Systems Engineering*.

[29] Engeberg E. (2013). A physiological basis for control of a prosthetic hand. *Biomedical Signal Processing and Control*.

[30] Slotine J., Li W. (2002). *Applied Nonlinear Control*.

[31] Werremeyer M., Cole K. (1997). Wrist action affects precision grip force. *Journal of Neurophysiology*.

[32] Netter F. (1998). *Atlas of Human Anatomy*.

[33] Matulevich B., Loeb G. E., Fishel J. A. Utility of contact detection reflexes in prosthetic hand control.

[34] Connolly C. (2008). Prosthetic hands from touch bionics. *Industrial Robot*.

[35] Kuiken T., Li G., Lock B. (2009). Targeted muscle reinnervation for real-time myoelectric control of multifunction artificial arms. *Journal of the American Medical Association*.

[36] Johannes M. S., Bigelow J. D., Burck J. M., Harshbarger S. D., Kozlowski M. V., Van Doren T. (2011). An overview of the developmental process for the modular prosthetic limb. *Johns Hopkins APL Technical Digest*.

[37] Kent B. A., Engeberg E. D. (2014). Human-inspired feedback synergies for environmental interaction with a dexterous robotic hand. *Bioinspiration and Biomimetics*.

[38] Kent B., Lavery J., Engeberg E. (2014). Anthropomorphic control of a dexterous artificial hand via task dependent temporally synchronized synergies. *Journal of Bionic Engineering*.

